# Self-Reported Practices and Emotions in Prescribing Opioids for Chronic Noncancer Pain: A Cross-Sectional Study of German Physicians

**DOI:** 10.3390/jcm11092506

**Published:** 2022-04-29

**Authors:** Erika Schulte, Frank Petzke, Claudia Spies, Claudia Denke, Michael Schäfer, Norbert Donner-Banzhoff, Ralph Hertwig, Odette Wegwarth

**Affiliations:** 1 Department of Palliative Medicine, Universitätsmedizin Göttingen, 37075 Göttingen, Germany; 2 Pain Clinic, Department of Anesthesiology, Universitätsmedizin Göttingen, 37075 Göttingen, Germany; frank.petzke@med.uni-goettingen.de; 3 Department of Anesthesiology and Operative Intensive Care Medicine (CCM, CVK), Charité—Universitätsmedizin Berlin, Corporate Member of Freie Universität Berlin and Humboldt-Universität zu Berlin, and Berlin Institute of Health, 13353 Berlin, Germany; claudia.spies@charite.de (C.S.); claudia.denke@charite.de (C.D.); micha.schaefer@charite.de (M.S.); odette.wegwarth@charite.de (O.W.); 4 Department of Primary Care, Phillips-Universität Marburg, 35043 Marburg, Germany; norbert@staff.uni-marburg.de; 5 Center for Adaptive Rationality, Max-Planck-Institut für Bildungsforschung, 14195 Berlin, Germany; hertwig@mpib-berlin.mpg.de

**Keywords:** chronic noncancer pain, WHO III opioids, prescription, guideline adherence

## Abstract

Background: The pressure on physicians when a patient seeks pain relief and their own desire to be self-effective may lead to the prescription of strong opioids for chronic noncancer pain (CNCP). This study, via physician self-reporting, aims to identify and measure (i) physician adherence to national opioid prescribing guidelines and (ii) physician emotions when a patient seeks a dosage increase of the opioid. Methods: Within a cross-sectional survey—conducted as part of a randomized controlled online intervention trial (ERONA)—600 German physicians were queried on their opioid prescribing behavior (choice and formulation of opioid, indications) for CNCP patients and their emotions to a case vignette describing a patient seeking an opioid dosage increase without signs of objective deterioration. Results: The prescription of strong opioids in this study was not always in accordance with current guidelines. When presented with a scenario in which a patient sought to have their opioid dose increased, some physicians reported negative feelings, such as either pressure (25%), helplessness (25%), anger (23%) or a combination. The risk of non-guideline-compliant prescribing behavior using the example of ultrafast-acting fentanyl for CNCP was increased when negative emotions were present (OR: 1.7; 95%-CI: 1.2–2.6; *p =* 0.007) or when sublingual buprenorphine was prescribed (OR: 15.4; 95%-CI: 10.1–23.3; *p* < 0.001). Conclusions: Physicians’ emotional self-awareness represents the first step to identify such direct reactions to patient requests and to ensure a responsible, guideline-based opioid prescription approach for the long-term well-being of the patient.

## 1. Introduction

Strong opioids have been used for chronic noncancer pain (CNCP) since the 1980s [1]. While opioids show low-to-moderate improvements in pain relief and functionality, the administration of opioids in CNCP can have relevant side effects [2,3,4,5,6,7], including opioid use disorders [8,9,10]. Therefore, international and national guidelines have been brought into place [11,12,13] to help balance the benefits and risks, increase safety and inform prescribers about the evidence-based use of opioids in CNCP.

However, a current review shows that physicians’ adherence to the North American Opioid Guidelines and their compliance with the recommendations has been low: treatment agreements, consented urine drug testing, consultations with drug monitoring programs, assessing the risk of aberrant medication-taking behavior and mental health screening [14]. To the best of our knowledge, we are not aware of any studies that have specifically focused on physicians’ guideline adherence with regard to the indications for opioids and the individual opioid formulations. This gave rise to our motivation to investigate the extent to which physicians in Germany prescribe strong opioids for CNCP in line with the indication and the drug formulation.

Despite the lack of evidence for an opioid crisis in Germany comparable to the one in the US [15,16], there remains considerable uncertainty [13] as to when and for how long the types and formulations of opioids should be prescribed for CNCP, leaving ample responsibility with the treating physician. National guidelines have outlined recommendations to support the decision-making process prior to the initiation of opioid treatment that should integrate both the evaluation of risks and the potential benefits of opioids [17]. To date, opioid risk mitigation programs have focused primarily on the individual patient and on preventing addiction, misuse and overuse of opioids [18] and less on physicians’ prescriptions habits and the challenges they may face when responding to a patient’s desire for pain reduction through opioids and overall limited therapy options to treat chronic pain.

Studies focusing on the physician’s decision process show, for example, that they prescribe more opioids to more psychologically stressed patients who express their suffering clearly [19]. In a qualitative study, opioid-prescribing general practitioners expressed substantial frustration and stress in managing chronic pain patients with opioids [20]. However, most studies focused on the patient characteristics (for example, consistent and objective information given by the patient, red flags, patient trustworthiness) that lead the provider to prescribe opioids for CNCP [21,22,23,24]. We could not find a single study investigating in depth the “inner conflict” of the opioid prescribing physician in Germany. Therefore, we tried to find phrases that describe this inner conflict and interviewed physicians regularly prescribing opioids for CNCP about whether they can identify themselves with these phrases of “inner conflict”.

Taken together, the present analysis examined (I) the adherence of physicians in Germany to the national guideline recommendations for long-term opioid use with regard to indications and formulations of strong opioids in CNCP, (II) physicians’ emotional reactions to patients’ requests for an increased opioid dose without objective worsening of the underlying condition (presented as a case study vignette) and (III) possible prescriber-related factors that might be associated with non-guideline-compliant prescriptions.

## 2. Methods

The ERONA (experiencing the risk of overutilizing opioids among patients with chronic noncancer pain in ambulatory care) project—consisting of four prospective exploratory, randomized controlled online trials (RCT) with four independent study populations—aimed to investigate experiential versus text-based educational formats (DRKS00020358). The full peer-reviewed study protocol is published elsewhere [25]. The data reported here are based on survey questions that were included in the RCT prior to randomization of the physicians to one of two educational interventions addressing the benefit-to-harm ratio of strong opioids, defined as World Health Organization Step III opioids.

### 2.1. Study Population and Inclusion Criteria

Using a multi-layered strategy, IPSOS Health, an independent market research institution, recruited randomly selected potentially eligible physicians via its panels and business directories (e.g., directory of the National Association of Statutory Health Insurance Physicians). The first contact was made by e-mail. Interested physicians were then contacted by phone. The final intervention was online. All participants were reimbursed for participation by IPSOS Health. To detect a 15% difference in the randomized ERONA trial, 300 participants per intervention arm were recruited between April 2020 and August 2020, resulting in a sample of 600 physicians over a wide range of disciplines. Details of the sample size calculation were already published [25]. Only physicians prescribing strong opioids regularly for CNCP were included in the study and detected by the screener question: “Do you prescribe BtM (Betäubungsmittel)-based opioids to treat patients with chronic, non-tumor-related pain?”. For strong opioids in Germany, it is necessary to make the prescription on a special prescription form, called *BtM*-prescription. Informed consent was obtained online prior to the study.

### 2.2. Survey Questionnaire

#### 2.2.1. Baseline Characteristics

The following physician baseline characteristics were recorded in the online survey: age, gender, region of work (north, south, east, west of Germany), years of working experience, and workplace (doctor’s office, medical care center, hospital, rehabilitation clinic/nursing home).

#### 2.2.2. Opioid-Prescribing Behavior: Type and Formulation of Opioids, Indications

Physicians were asked about their opioid-prescribing behavior with two questions: (1) “Under which noncancer-related chronic pain conditions have you prescribed strong opioids for as the primary prescriber within the past 12 months?” and (2) “Which of the following strong opioid formulations are you currently prescribing for the treatment of chronic noncancer pain”? Regarding question 1, the list of suggested diagnoses was “chronic nonspecific low-back pain, osteoarthrosis, diabetic polyneuropathy, postherpetic neuralgia, phantom limb pain, disc prolapse, spinal stenosis, rheumatoid arthritis, fibromyalgia syndrome, secondary headaches, osteoporotic vertebral body fractures, chronic postsurgical pain, peripheral artery disease of the lower extremities, grade 3 and 4 pressure ulcers, chronic pain associated with fixed contractures, central neuropathic pain, chronic regional pain syndrome I and II, chronic pelvic pain associated with adhesions or endometriosis, chronic inflammatory bowel disease, primary headaches, functional disorders, chronic pancreatitis, craniomandibular dysfunction, persistent idiopathic facial pain, neuralgia (e.g., trigeminus) and multiple sclerosis” with the answer options “Yes/No/Does not apply”. “Does not apply” was explained with “I haven’t had a patient with this type of chronic pain condition.”.

Regarding question 2, the list of selectable opioids included “morphine, buprenorphine, fentanyl, oxycodone, hydromorphone and tapentadol”, each supplemented with the most common German trade names and the following prescribing options currently available in the German national formulary: “oral extended release/oral (or nasal or sublingual) immediate release/transdermal/or I do not prescribe this strong opioid at all”. The opioids and their possible formulations were presented as a table with mandatory fields—excluding non-available combinations, such as transdermal and morphine. “Noncancer-related chronic pain condition” or “chronic noncancer pain” was printed in bold in question 1 and 2 of the survey, respectively. The answers to the indication questions were based on the German guideline recommendations for long-term use of opioids in chronic noncancer pain (LONTS) [13]. This guideline defines both evidence-based as well as consensus-based indications and contraindications for opioid therapy and was published in its current version prior to the survey.

#### 2.2.3. Physicians’ Emotional Response to Patients’ Demands for Dose Escalation

Each physician was further presented a case vignette in which a patient with nonspecific low back pain and longstanding opioid therapy asked for an increase in opioid dose even though there was no evidence of objective somatic deterioration. The exact wording of the vignette and the respective question are given in Table 4. The five options for a response on an emotional level can also be found in Table 4. “Yes” or “no” was the possible answer for each statement. All statements needed to be affirmed or denied.

#### 2.2.4. Risk Literacy

The physicians’ medical risk literacy was assessed by administering an adapted version of the validated Critical Risk Interpretation Test (CRIT) [26]. The score of correct responses ranged from 0 to 5 with the latter being the highest possible degree of risk literacy.

#### 2.2.5. Piloting

The questionnaire used in this study was piloted with 11 physicians that regularly treated CNCP patients: general practitioners and pain specialists with varying degrees of experience, both in the outpatient and in the hospital setting. They answered the questions as study participants, and they were also asked to give comments on the comprehensibility and quality of the questions. With their feedback, the framing and wording of the survey questions were revised and optimized. Both the German original version of the questions analyzed here and the English translation can be viewed in the Supplementary Materials.

### 2.3. Statistical Analysis

The survey did not permit any non-responses to the questionnaire items; thus, all datasets were complete. The data were descriptively analyzed by frequency distributions and percentages. A binary logistic regression model was used to explore potential associations between non-guideline compliant opioid prescription behavior—using the prescription of oral/nasal ultrafast acting fentanyl for CNCP as an example—and independent variables that may affect prescriptions, such as age, gender, work experience, prescription of other substances, such as buprenorphine, and the presence of negative emotions (at least one of the four possible suggested negative emotions). For the insertion of the independent variables into the model, the forward stepwise method was used. *p* < 0.05 was considered significant. Data were stored and analyzed with IBM SPSS Statistics (version 27) (Armonk, NY, USA).

## 3. Results

### 3.1. Recruitment of Participating Physicians

**Figure 1:**
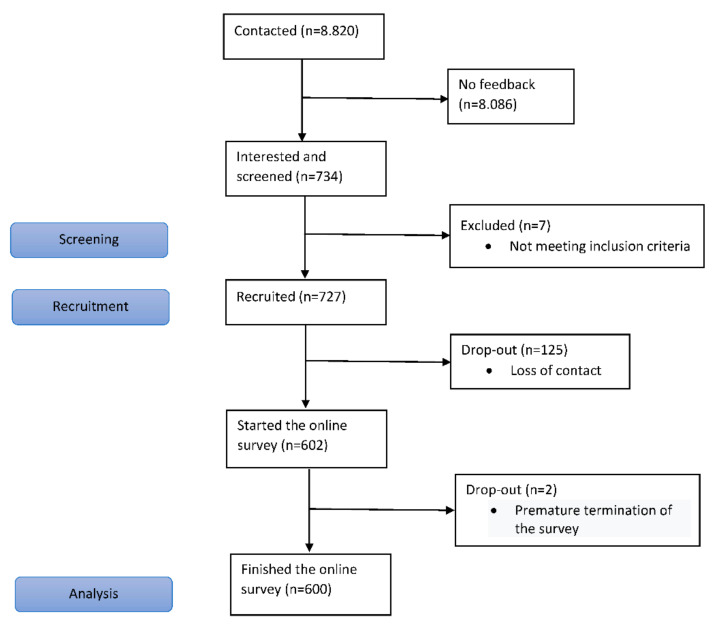
Flow chart of the study.

IPSOS contacted successfully contacted 8820 physicians. Of the 734 physicians who were interested in taking part in the survey, 7 did not meet the screener criterion, i.e., regularly prescribing opioids for CNCP; thus, 727 were recruited for the survey. A further 125 physicians, who had originally agreed to participate, eventually chose to not take part in the survey. Of the remaining 602 physicians who started the survey, 2 left the survey prematurely and 600 completed the survey. In the end, 6.8 percent of the contacted physicians answered the survey (Figure 1).

### 3.2. Demographic and Professional Characteristics

The proportion of men among the physicians surveyed was 69%. Most of the respondents (69%) were middle-aged (40–59 years of age), resulting in a huge group of participants (74%) with around 10–30 years of professional experience. The largest category of physicians who regularly prescribed opioids for CNCP according to this survey were general practitioners (60%), followed by specialist internists (25%), anesthetists (11%) and orthopedists (6%). All other specialist groups were equal to or less than 2% (Table 1). Most of the physicians worked in their own practice (Table 1).

### 3.3. Self-Reports of the Opioid Ingredients and Galenics Prescribed

Transdermal fentanyl (99%), slow-release morphine (98%) and slow-release oxycodone (91%) were reported to be prescribed most frequently for CNCP in this survey. Of the 600 participants, 41% and 49% prescribed sublingual forms of buprenorphine and the ultrafast acting application of fentanyl (oral or nasal), respectively. The frequency of the other prescribed opioids for CNCP is shown in Table 2.

### 3.4. The Indications Detailed in the Self-Reported Opioid Prescribing Behavior

Physicians most often reported that their opioid prescription was for the following diagnoses: disc prolapse (62%) and grade 3 and 4 pressure ulcers (60%); the national LONTS guideline specifies an open recommendation for these (Table 3). Osteoarthritis was the most common indication (56%) that physicians reported prescribing strong opioids, for which the LONTS guideline provides an evidence-based positive recommendation for short-term (4–12 weeks) and immediate-term (13–26 weeks) use (Table 3). For chronic nonspecific low back pain, 38% of physicians reported prescription rates of strong opioids, for diabetic polyneuropathy 41% and for postherpetic neuralgia 38%, which follows positive (short-term use) or open guideline recommendations (long-term use) (Table 3). Although for the following diagnosis, the LONTS recommendations are negative (Table 3), 42% of physicians reported prescribing strong opioids in chronic inflammatory bowel disease, 30% in chronic pancreatitis, 26% in functional disorders, 25% in fibromyalgia syndrome and 20% in primary headaches.

### 3.5. Physicians’ Self-Reported Emotional Reactions to Patient Requests to Increase Opioid Dosages

**Figure 2:**
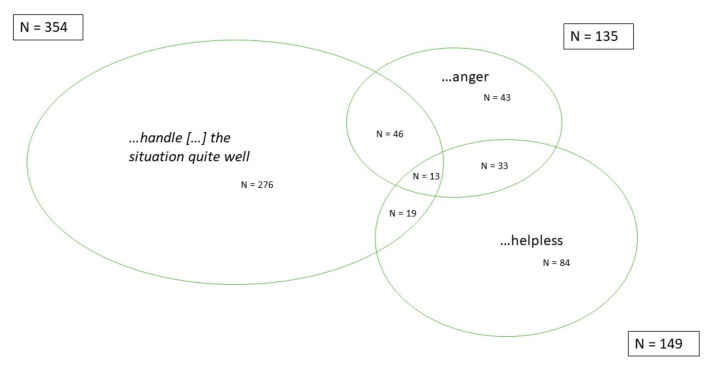
A selection of physician self-reported emotional reactions to a patient’s desire to increase opioid dosage and their overlaps.

The feeling of being well-equipped to handle a patient’s request of increasing the opioid dosage to treat unspecific low-back pain was reported by 59% of the physicians, whereas 43% of the physicians described negative feelings in such a situation. About one quarter of the physicians expressed feelings of either pressure (25%), helplessness (25%), anger, or a combination (23%) (Table 4). A smaller subgroup of physicians reported that despite negative feelings they can handle the situation quite well: 59 physicians reported anger and good management of the situation (10% of the whole group); helplessness and good management of the situation was reported by 32 physicians (5% of the whole group) (Figure 2).

### 3.6. Covariate Analysis of Non-Guideline-Compliant Opioid Prescribing Behavior

Physicians prescribing ultrafast-acting fentanyl formulations were also highly likely to prescribe sublingual buprenorphine (OR: 15.4; 95%-CI: 10.1–23.3; *p* < 0.001). The presence of negative emotions in response to patients’ demands for a dose escalation nearly doubled the likelihood of physicians to prescribe ultrafast-acting fentanyl to their patients (OR: 1.7; 95%-CI: 1.2–2.6; *p =* 0.007). Other aspects, such as work experience or risk literacy, were not found to be associated with physicians’ prescription behavior. The final independent variables included in the binary logistic regression analysis (Table 5) increased the proportion of correctly predicted answers from 51.0% to 78.5%. The selected model explains 41% of the existing variance (Nagelkerkes R-Quadrat).

## 4. Discussion and Conclusions

This analysis of the ERONA study showed that physicians in Germany reported a prescribing behavior for strong opioids for CNCP that was not consistently compliant with current guidelines—both in terms of opioid indications and the opioid formulations selected. A bad feeling about increasing an opioid dose in a situation without objective signs of deterioration was reported by 43% of the physicians surveyed. Emotions such as pressure, helplessness and anger were reported by 25%, 25% and 23% of the physicians in this situation. Perceived negative feelings about an opioid increase were associated with more non-compliant prescribing behavior.

The LONTS guideline, based on controlled clinical studies, recommends opioid prescription at least for 4–12 weeks for the following four diagnoses: chronic nonspecific low back pain [27], osteoarthrosis [28], diabetic polyneuropathy and postherpetic neuralgia [29]. The prescribing behavior of the physicians surveyed in our study indicated that they prescribe strong opioids most frequently for osteoarthritis (OA) (56%), and less often for chronic nonspecific low back pain (CLBP) (38%) (Table 3). This corresponds to the recommendation of the National Guideline for CLBP [30], which specifies opioids may be a short-term option only for selected patients. Comparing treatment guidelines for OA and CLBP, psychosocial factors seem to be more prominent in CLBP than in OA, where they certainly also play a role, but the somatic pain component is usually in the foreground [31,32]. In addition, OA pain often affects older people, where other therapy options, such as NSAIDs, are often either contraindicated [33], exercise therapy is more limited [34], or both.

The opioid prescriptions by physicians in this study for functional disorders, fibromyalgia syndrome and primary headaches appeared problematic, and they were anticipated to have negative consequences for the patient, such as unwanted medication overuse, headache (MOH) [12] or problematic opioid use. Prevalence data on the use of opioids in functional disorders are difficult to define since various heterogeneous diseases are collated under this diagnosis.

Further, immediate-release opioids are seldom necessary for noncancer pain [13] and ultrafast-acting opioids are exclusively licensed for cancer pain [35]. The fact that we found 49% of physicians in our study prescribing ultrafast-acting fentanyl to CNCP can be considered highly problematic. The challenge of non-indicated “off-label-use” has been described previously [36,37] and has been observed in other cohorts, for instance among Italian patients with CNCP of which nearly 10% received ultrafast onset opioids [38]. Training, education and the implementation of prevention strategies—possibly including either medico-legal consequences, non-reimbursement by the health insurance companies or both—would be necessary measures to restrict such misuse [39].

The role of sublingual buprenorphine, on the other hand, must be seen in a differentiated manner. It has a significantly longer onset and action time than fast-acting fentanyl. It is also approved for non-cancer pain, unlike the rapid-acting fentanyl preparations. In this respect, we were surprised by the fact that physicians who prescribe sublingual buprenorphine, which can be indicated in CNCP, were 15 times more likely to also prescribe fast-acting fentanyl, which is not indicated in CNCP. Perhaps these figures expressed the fact that those physicians were well versed in handling a wide variety of preparations and galenics. However, even within this group, there were those who adhere to the indications and others that do not.

In our study, 43% of the physicians reported a negative emotion associated with their own responsibility to a dose elevation in response to a patient’s demand to increase opioid dosage without any obvious deterioration. Our study documented—to the best of our knowledge, for the first time—that such negative emotions may significantly influence physicians’ reaction to these demands: Physicians presenting with negative emotions tended to exemplify more non-guideline-compliant behavior than did those who did not report such negative emotions. Not having negative emotions appeared to protect physicians from prescribing and patients from receiving an opioid medication that is not indicated for noncancer chronic pain. These findings on the role of negative emotions on patients’ potentially unwarranted demand for dosage increase has been seldom described until now. However, it was in line with the prescriber style described by Passik et al., which is, for example, characterized by “aggressive opioid titration […] with intents to entirely eliminate pain.” [40]. It is of course also conceivable that negative gut feelings warn against an unjustified prescription.

Considering the consequences of non-compliant prescriptions of potent but risky drugs for patients’ safety, the currently rather under-studied influence of different negative emotional states on physicians’ compliance to guidelines certainly requires more research on these aspects to inform curricula and continuing training programs on appropriate opioid prescriptions.

Our study had some limitations. A critical point of this study was the high non-response rate during the recruitment process because the remaining physician cohort may not be representative of physicians working in Germany. At first glance, for example, women seemed to be underrepresented. However, if one compares the proportion of women in our study with the data of the German Medical Association (GMA) [41] based on the six most frequently occurring groups of expertise in this study, the proportion is 37% compared to 40% (GMA), thus only a little bit lower. If it was considered that older and more experienced physicians answered this survey, our data appeared to be quite representative in this point since the proportion of women is lower in the group of older physicians. The external validity of this study of course remains a critical point. For example, physicians may have responded who are already more critical of their prescription of opioids or more aware of their feelings than others. However, the focus of this study should be to draw attention to the presence of emotional aspects of opioid prescription and not to claim that the numbers determined were absolutely correct.

Another limitation was that these data are only based on self-reports, which may invite inaccuracies due to social desirability. Questions may have been misunderstood, e.g., that the addition “for noncancer pain” was missed in the questions about opioid preparations. We hoped to have minimized such issues by piloting material with colleagues. A further limitation is that we did not know how frequently non-guideline-compliant prescriptions occurred, as we have no information about the patients actually seen in these practices and the rates of prescription. Another inherent limitation was that the current study showed only an association between negative emotions and prescribing behavior rather than a cause-and-effect relationship. It could be, for example, that physicians’ overprescribing was leading to a feeling of helplessness and anger, but it could also be that their initial feeling of anger and helplessness was leading to overprescribing. Other study designs, especially either qualitative designs, longitudinal quantitative designs or a combination, should investigate these questions. Another important point is that the questions concerning the emotional reactions of the physicians are not a part of a validated questionnaire. This reduces the internal validity of the results. After a qualitative scientific examination of the topic “physicians’ emotions and prescribing opioids” as suggested above, the goal should be to generate a validated questionnaire that serves both research purposes and routine use to sensitize physicians to this topic.

This analysis showed that in Germany strong opioids are largely prescribed in accordance with the existing guidelines. However, there were indications where the use of opioids should be viewed critically, e.g., in the case of primary headaches, fibromyalgia syndrome or other functional pain syndromes. Fast-acting fentanyl preparations should not be used in CNCP. Emotional aspects on the part of the prescribing physician could also play a role if opioids are not prescribed in accordance with the guidelines.

## Figures and Tables

**Table 1:** Demographic and professional characteristics of the physicians surveyed.

	**Total**
	N = 600
	N (%)
Gender	
female	221 (36.8)
Age (years)	
20–39	51 (8.5)
40–59	413 (68.8)
60–79	136 (22.7)
Place of work in Germany	
North	133 (22.2)
South	133 (22.2)
East	160 (26.7)
West	174 (29.0)
Work experience (years)	
<10	46 (7.7)
10–19	199 (33.2)
20–29	247 (41.2)
>30	108 (18.0)
Type of workplace	
Doctor’s office	386 (64.3)
Medical care center	149 (24.8)
Hospital	58 (9.7)
Rehabilitation clinic/nursing home	7 (1.2)
Areas of expertise ^a^	
General medicine	360 (60.0)
Internal medicine	149 (24.8)
Anesthesiology	68 (11.3)
Orthopedic surgery	40 (6.7)
Psychiatry/psychotherapy/psychosomatic	12 (2.0)
Neurology	11 (1.8)
General surgery	11 (1.8)
Physical medicine	3 (0.5)
Gynecology	1 (0.2)
Urology	1 (0.2)

^a^ Sum of expertise fields is >600 because some physicians have more than one specialization.

**Table 2:** Self-reported opioid prescribing behavior: opioid variant and formulations.

*“**Which of the following Strong Opioids** are you Currently Prescribing for the Treatment of **Chronic Noncancer Pain*** *and in which Dosage Form?”*	**Total**
	N = 600
	N (%)
Morphine	
Oral extended release	587 (97.8)
Oral immediate release	517 (86.2)
No use	9 (1.5)
Buprenorphine	
Transdermal	482 (80.3)
Sublingual	245 (40.8)
No use	102 (17.0)
Fentanyl	
Transdermal	594 (99.0)
Oral/nasal immediate release	294 (49.0)
No use	2 (0.3)
Oxycodone	
Oral extended release	545 (90.8)
Oral immediate release	468 (78.0)
No use	5 (0.8)
Hydromorphone	
Oral extended release	473 (78.8)
Oral immediate release	207 (34.5)
No use	117 (19.5)
Tapentadol	
Oral extended release	515 (85.8)
Oral immediate release	354 (58.3)
no use	51 (8.5)

**Table 3:** Indications of physician self-reported opioid prescribing behavior compared to guideline recommendations.

*“For which **noncancer-related diseases** have you prescribed strong opioids as the primary prescriber in the past 12 months?”*	**Total**	Evidence Level According to LONTS ^b^ [13]
	N = 600	
	N (%)	
***Chronic nonspecific low-back pain***		
Yes	225 (37.5)	4–12 weeks: Ia, recommendation for
No	335 (55.8)	13–26 weeks: Ia, recommendation for
Does not apply ^a^	40 (6.7)	>26 weeks: IIb, open recommendation
***Osteoarthritis***		
Yes	335 (55.8)	4–12 weeks: Ia, recommendation for
No	238 (39.7)	13–26 weeks: Ia, recommendation for
Does not apply ^a^	27 (4.5)	>26 weeks: IIb, open recommendation
***Diabetic polyneuropathy***		
Yes	248 (41.3)	4–12 weeks: Ia, strong recommendation for
No	210 (35.0)	13–26 weeks: no data, open recommendation
Does not apply ^a^	142 (23.7)	>26 weeks: IIb, open recommendation
***Postherpetic neuralgia***		
Yes	229 (38.2)	4–12 weeks: Ia, recommendation for
No	273 (45.5)	13–26 weeks: no data, open recommendation
Does not apply	98 (16.3)	>26 weeks: no data, open recommendation
***Phantom limb pain***		
yes	289 (48.2)	4–12 weeks: Ib, open recommendation for
no	186 (31.0)	13–26 weeks: no data, open recommendation
does not apply ^a^	125 (20.8)	>26 weeks: no data, open recommendation
***Disc prolapse*** ^c^		
yes	370 (61.7)	4–12 weeks: Ib, open recommendation for
no	200 (33.3)	13–26 weeks: no data, open recommendation
does not apply ^a^	30 (5.0)	>26 weeks: no data, open recommendation
***Spinal stenosis***		
yes	251 (41.8)	4–12 weeks: Ib, open recommendation for ^c^
no	287 (47.8)	13–26 weeks: no data, open recommendation ^c^
does not apply ^a^	62 (10.3)	>26 weeks: no data, open recommendation ^c^
*****Rheumatoid arthritis*****		
yes	263 (43.8)	4–12 weeks: Ib, open recommendation for
no	298 (49.7)	13–26 weeks: no data, open recommendation
does not apply ^a^	39 (6.5)	>26 weeks: no data, open recommendation
***Fibromyalgia syndrome***		
yes	152 (25.3)	4–12 weeks: Ib, open recommendation for
no	252 (42.0)	13–26 weeks: no data, open recommendation
does not apply ^a^	196 (32.7)	>26 weeks: no data, open recommendation
***Secondary headaches***		
yes	136 (22.7)	4–12 weeks: no data, open recommendation
no	380 (63.3)	13–26 weeks: no data, open recommendation
does not apply ^a^	84 (14.0)	>26 weeks: no data, open recommendation
***Vertebral body fractures in osteoporosis***		
yes	231 (38.5)	4–12 weeks: no data, open recommendation
no	268 (44.7)	13–26 weeks: no data, open recommendation
does not apply ^a^	101 (16.8)	>26 weeks: no data, open recommendation
***Chronic postsurgical pain***		
yes	336 (56.0)	4–12 weeks: no data, open recommendation
no	143 (23.8)	13–26 weeks: no data, open recommendation
does not apply ^a^	121 (20.2)	>26 weeks: no data, open recommendation
***Peripheral arterial disease of the lower extremities***		
yes	171 (28.5)	4–12 weeks: no data, open recommendation
no	337 (56.2)	13–26 weeks: no data, open recommendation
does not apply ^a^	92 (15.3)	>26 weeks: no data, open recommendation
***Grade 3 and 4 pressure ulcers***		
yes	362 (60.3)	4–12 weeks: no data, open recommendation
no	158 (26.3)	13–26 weeks: no data, open recommendation
does not apply ^a^	80 (13.3)	>26 weeks: no data, open recommendation
***Chronic pain associated with fixed contractures***		
yes	231 (38.5)	4–12 weeks: no data, open recommendation
no	254 (42.3)	13–26 weeks: no data, open recommendation
does not apply ^a^	115 (19.2)	>26 weeks: no data, open recommendation
***Central neuropathic pain***		
yes	112 (18.7)	4–12 weeks: no data, open recommendation
no	327 (54.5)	13–26 weeks: no data, open recommendation
does not apply ^a^	161 (26.8)	>26 weeks: no data, open recommendation
***Chronic regional pain syndrome I and II***		
yes	274 (45.7)	4–12 weeks: no data, open recommendation
no	172 (28.7)	13–26 weeks: no data, open recommendation
does not apply ^a^	154 (25.7)	>26 weeks: no data, open recommendation
***Chronic pelvic pain***		
yes	73 (12.2)	4–12 weeks: no data, open recommendation
no	217 (36.2)	13–26 weeks: no data, open recommendation
does not apply ^a^	310 (51.7)	>26 weeks: no data, open recommendation
***Chronic inflammatory bowel disease***		>26 weeks: IIIb, recommendation against
yes	252 (42.0)	
no	248 (41.3)	
does not apply ^a^	100 (16.7)	
***Primary headaches***		>26 weeks: IIIb, strong recommendation against
yes	119 (19.8)	
no	406 (67.7)	
does not apply ^a^	75 (12.5)	
***Functional disorders***		no data; independent of time: strong recommendation against
yes	158 (26.3)	
no	366 (61.0)	
does not apply ^a^	76 (12.7)	
***Chronic pancreatitis***		>26 weeks: IIIb, strong recommendation against
yes	180 (30.0)	
no	316 (52.7)	
does not apply ^a^	104 (17.3)	
***Craniomandibular dysfunction***		no recommendation
yes	86 (14.3)	
no	259 (43.2)	
does not apply ^a^	255 (42.5	
***Persistent idiopathic facial pain***		no recommendation
yes	197 (32.8)	
no	264 (44.0)	
does not apply ^a^	139 (23.2)	
***Neuralgia (e.g., trigeminus)***		no recommendation
yes	241 (40.2)	
no	295 (49.2)	
does not apply ^a^	64 (10.7)	
***Multiple sclerosis***		No statement on this indication in LONTS ^b^
yes	136 (22.7)	
no	295 (49.2)	
does not apply ^a^	169 (28.2)	

^a^ does not apply = physician has not treated patients with this disease in the past 12 months; ^b^ German guideline for long-term use of opioids in chronic noncancer pain; ^c^ recommendation for radiculopathy.

**Table 4:** Physician self-reported emotional reactions to a patient’s desire to increase opioid dosage in long-term opioid therapy of chronic unspecific low back pain.

**Case Vignette:** *“Please Imagine the Following Situation: A Patient with Chronic Noncancer Related Low-Back Pain who has Already been Prescribed an Opioid for a Long Time Comes to your Consultation with the Request to Increase the Opioid Dose. There are no Indications of a Finding that Requires Intervention, such as a New Neurological Disorder or Other red Flags.* ***Which of the Emotions Described below have you Already Observed in Yourself?”***	**Total**
	N = 600
	N ^a^ (%)
*“I can handle the situation **quite well**.”*	354 (59)
*“I feel **pressured** to increase the dose.”*	148 (25)
*“I feel **helpless** because I don’t have an easy solution.”*	149 (25)
*“I experience negative emotions such as **anger**.”*	135 (23)
*“I have a **bad feeling** about increasing the dose.”*	258 (43)

^a^ multiple answers were possible.

**Table 5:** Binary logistic regression analysis with non-guideline-compliant opioid prescribing (ultrafast-acting fentanyl for CNCP) as dependent variable.

	Non-Guideline-Compliant Opioid Prescribing Using the Example of Ultra-Fast-Acting Fentanyl for CNCP		
Independent Variables	Odds Ratio	95% CI	*p*
Buprenorphine, sublingual Prescribing for CNCP (reference class: no prescribing for CNCP)	15.4	10.1–23.3	<0.001
Negative emotions ^a^ present (reference class: not-present)	1.7	1.2–2.6	0.007

^a^ as reaction to the case study vignette relating to a patient request for an opioid dose increase.

## Supplementary Materials

The following supporting information can be downloaded at: https://www.mdpi.com/article/10.3390/jcm11092506/s1, Project: ERONA—Physicians.
