# Supplementary material for: Self-Reported Practices and Emotions in Prescribing Opioids for Chronic Noncancer Pain: A Cross-Sectional Study of German Physicians
Source: J Clin Med. 2022 Apr 29;11(9):2506. doi: 10.3390/jcm11092506 (PMC9104176; doi:10.3390/jcm11092506)
Supplement: Supplementary file 1 [file jcm-11-02506-s001.zip › jcm-1689437-supplementary.pdf]

Supplementary

Projekt: ERONA - Ärzte

[Note: Der ERONA Fragebogen bestand aus Fragen zu mehreren Teilprojekten. Nachfolgend sind nur die Fragen gelistet, die die aktuelle Studie betreffen.]

Liebe Ärztinnen und Ärzte,

vielen Dank für Ihr Interesse an dieser Studie.

Ipsos ist ein unabhängiges Marktforschungsinstitut. Im Auftrag des Max-Planck-Instituts für Bildungsforschung führen wir das Studienprojekt „ERONA“ (Experiencing the Risks of Overutilizing Opioids Among Patients with Non-Tumor Chronic Pain in Ambulant Care) – gefördert durch das Bundesministerium für Gesundheit – durch. Das Projekt beabsichtigt, besser zu verstehen, wie Ärztinnen und Ärzte Behandlungsentscheidungen zu starken Opioiden treffen und wie sie deren Wirkung sehen.

Die Erkenntnisse aus diesem Projekt sollen zukünftig dabei helfen, besser einschätzen zu können, wie medizinische Risiken dargestellt werden sollten, damit sie ärztliche Behandlungsentscheidungen bestmöglich unterstützen. Das Max-Planck-Institut würde sich daher glücklich schätzen, wenn Sie an der Online-Befragung teilnehmen. Die Befragungsdauer beträgt in etwa **30 Minuten** und ist incentiviert. Bevor Sie beginnen, möchte wir Sie noch einmal darauf hinweisen, dass Ihre Teilnahme absolut freiwillig ist. Sie können die Teilnahme zu jedem Zeitpunkt ohne Angabe von Gründen und ohne etwaige Nachteile beenden.

Wir möchten Ihnen Folgendes versichern:

- *Wir werden alle in Deutschland gültigen Gesetze zum Schutz Ihrer personenbezogenen Daten sowie die Leitlinien der Europäischen Gesellschaft für Meinungs- und Marktforschung e.V. (ESOMAR) und des Arbeitskreises Deutscher Markt- und Sozialforschungsinstitute e.V. (ADM) befolgen. Die Datenschutzerklärung für diese Studie erhalten Sie* hier.
- *Ihre Antworten werden sowohl von uns als auch vom auftraggebenden Unternehmen nur für Forschungszwecke verwendet.*
- *Ihre Antworten werden mit denen der anderen Teilnehmer zusammengefasst und dem Auftraggeber dieser Studie in aggregierter und anonymisierter Form vorgestellt.*

Wenn Sie an der Befragung teilnehmen wollen und mit den Bedingungen einverstanden sind, klicken Sie am Ende dieser Seite bitte auf „Ich stimme zu“. Ansonsten schließen Sie einfach die Befragung.

Durch die Befragung navigieren Sie, indem Sie am Ende jeder Seite die Schaltfläche „Weiter“ anklicken. Falls diese Schaltfläche nicht sichtbar ist, scrollen Sie bitte nach unten. Sollte es Ihnen nicht möglich sein, zur nächsten Seite überzugehen, haben Sie möglicherweise eine Frage auf der Seite übersehen. Suchen Sie in diesem Fall bitte nach einer Fehlermeldung und scrollen Sie nach unten.

Das Projekt ERONA ist ein Verbundprojekt des Max-Planck-Instituts für Bildungsforschung Berlin, der Charité – Universitätsmedizin Berlin, des Robert Koch-Instituts Berlin, und des Instituts für Evidenz in der Medizin (für Cochrane Stiftung Deutschland), Freiburg.

Wir danken Ihnen schon jetzt für Ihre Mithilfe.

Um mit unserer Studie möglichst aussagekräftige Ergebnisse zu erzielen, ist es wichtig, dass gezielt jene Personen befragt werden, die in der Realität am meisten von dem Untersuchungsthema betroffen sind. Die nachfolgenden Fragen – sogenannte Screener-Fragen – dienen der Identifikation dieser Personen. Bitte gehen Sie die Screener-Fragen Schritt für Schritt durch. In Abhängigkeit davon, ob Sie zu dem von uns gesuchten Personenkreis gehören, geht es nach diesen Fragen entweder direkt mit der eigentlichen Studie weiter für Sie, oder Ihre Teilnahme endet direkt danach.

S01 [S]

Welche der nachfolgenden Bezeichnungen beschreibt Ihre Tätigkeit?

1 Hausärztlich tätige/r Ärztin/Arzt
2 Fachärztin/-arzt mit Zusatzbezeichnung „Spezielle Schmerztherapie“
3 Ich bin weder hausärztlich tätig noch habe ich die Zusatzbezeichnung „Spezielle Schmerztherapie“.

S02 [O]

Welche Facharztdisziplin haben Sie?

____

S03 [S]

Verschreiben Sie zur Behandlung von Patienten mit chronischen, nichttumorbedingten Schmerzen BtM-pflichtige Opioide (z.B. Morphin, Sevredol®, Buprenorphin, Norspan®, Transtec®, Temgesic®, Fentanyl, Durogesic®, Oxycodon, Oxygesic®, Targin®, Hydromorphon, Palladon®, Jurnista®, Tapentadol, Palexia®)?

1 Ja
2 Nein

Herzlich willkommen bei der Studie ERONA! Bevor wir mit spezifischen Fragen rund um die Verordnung von WHO-III-Opioide im Kontext der Behandlung von Patient*innen mit chronischen, nichttumorbedingten Schmerzen beginnen, möchten wir als erstes einige demografische Aspekte erheben.

A01 [S]

Bitte geben Sie Ihr Geschlecht an.

1 weiblich
2 männlich
3 divers

A02 [S]

Bitte wählen Sie Ihre Altersklasse aus.

1 < 20 Jahre
2 20-39 Jahre
3 40-59 Jahre
4 60-79 Jahre
5 ≥ 80 Jahre

A03 [S]

Auf wie viele Berufsjahre blicken Sie bereits zurück?

1 < 10 Jahre
2 10-19 Jahre
3 20-29 Jahre
4 30-39 Jahre
5 ≥ 40 Jahre

A04 [S]

Wo arbeiten Sie hauptsächlich?

1 in einer Praxis
2 in einem medizinischen Versorgungszentrum
3 in einem Krankenhaus
4 in einer Reha-/Pflegeeinrichtung

A05 [S]

In welcher Region Deutschlands praktizieren Sie?

1 Nord [Schleswig-Holstein, Mecklenburg-Vorpommern, Niedersachsen, Hamburg, Bremen]
2 Süd [Bayern, Baden-Württemberg]
3 Ost [Sachsen-Anhalt, Brandenburg, Sachsen, Thüringen, Berlin]
4 West [Hessen, Nordrhein-Westfalen, Rheinland-Pfalz, Saarland]

B01 [M per row]

Lassen Sie uns nun die Versorgung von Patient*innen mit chronischen, nichttumorbedingten Schmerzen mit WHO-III-Opioiden genauer betrachten. Opioide können positive und negative Wirkungen auf die Gesundheit Ihrer Patient*innen haben. Nachfolgend sind einige der positiven und der negativen Wirkungen einer Behandlung mit starken Opioiden bei Patient*innen mit chronischem, nichttumorbedingten Schmerz aufgeführt. Bitte geben Sie für jede dieser Wirkungen an, ob Sie diese schon einmal persönlich bei Ihren Patient*innen beobachtet haben oder von diesen bisher nur durch Leitlinien, Fachartikel, Studienergebnisse o.Ä. gehört haben. Falls beides zutrifft, kreuzen Sie bitte beide Optionen an. Falls keine der Optionen zutrifft, kreuzen Sie bitte das Feld „keines davon" an. Wir bitten Sie also darum, in jeder Zeile ein Kreuz zu setzen.

Mögliche Wirkungen der Opioide	Das habe ich bei meinen Patienten bereits selbst beobachtet	Davon habe ich durch Leitlinien, Fachartikel, Studienergebnisse o.Ä. gehört	Keines davon
Reduktion der Schmerzen	☐	☐	○
Verbesserte Bewältigung des Alltags	☐	☐	○
Wiederaufnahme des Berufs	☐	☐	○
Verstärkte Müdigkeit	☐	☐	○
Verminderung der kognitiven Fähigkeiten	☐	☐	○
Auftreten von Schwindel	☐	☐	○
Auftreten von Stürzen	☐	☐	○
Verminderte Konzentrationsfähigkeit	☐	☐	○
Verminderte emotionale Schwingungsfähigkeit	☐	☐	○
Gefühl von Lustlosigkeit	☐	☐	○
Auftreten von Obstipation	☐	☐	○
Auftreten von Schwitzen	☐	☐	○
Auftreten von Übelkeit und Erbrechen	☐	☐	○
Schwierigkeiten beim Wasserlassen	☐	☐	○
Verminderte Libido	☐	☐	○
Kontinuierliche Abnahme der Wirkung der Opioide	☐	☐	○
Zunahme der Schmerzen	☐	☐	○
Bewusste oder unbewusste Einnahme zur emotionalen Spannungsreduktion	☐	☐	○
Anzeichen einer missbräuchlichen Einnahme	☐	☐	○

(intentional unsachgemäße Einnahme)			
Anzeichen einer fehlerhaften Einnahme (versehentlich unsachgemäße Einnahme)	☐	☐	○
Anzeichen einer Opioidabhängigkeit	☐	☐	○

Im Folgenden möchten wir Ihnen Fragen stellen, die dazu verleiten können, eine Antwort zu geben, von der man glaubt, sie träfe am ehesten auf soziale Zustimmung. Damit unsere Studie jedoch aussagekräftige Ergebnisse liefern kann, benötigen wir unverfälschte Daten. Ihre persönlichen Daten sind genau aus diesem Grunde mit einem Code verschlüsselt, damit Sie ohne Bedenken die Antwort geben können, die Ihre Meinung am besten widerspiegelt.

D01 [S per row]

Für **welche nichttumorbedingten Erkrankungen** haben Sie in den zurückliegenden 12 Monaten als Primärverordner*in WHO-III-Opioide verordnet? (Trifft nicht zu = hatte keine/n Patient/in mit diesem Krankheitsbild)

**Erkrankung**	**Ja**	**Nein**	**Trifft nicht zu**
Diabetische Polyneuropathie	○	○	○
Chronisch entzündliche Darmerkrankungen (z.B. M. Crohn, Colitis ulcerosa)	○	○	○
Clusterkopfschmerz	○	○	○
Craniomandibuläre Dysfunktion	○	○	○
Postzosterneuralgie	○	○	○
Dekubitus Grad 3 und 4	○	○	○
Arthrose	○	○	○
Unspezifischer Rückenschmerz	○	○	○
Funktionelle Störungen	○	○	○
Bandscheibenprotrusion, -prolaps	○	○	○
Fixierte Kontrakturen	○	○	○
Primäre Kopfschmerzen	○	○	○
Spinalkanalstenose	○	○	○
Manifeste Osteoporose mit Frakturen der Wirbelsäule	○	○	○
Chronische Pankreatitis	○	○	○

Phantomschmerz	○	○	○
Chronische postoperative Schmerzen	○	○	○
Chronic regional pain syndrome (CRPS) Typ I und II (M. Sudeck)	○	○	○
Endometriose	○	○	○
Gehirnläsion (z.B. Apoplex)	○	○	○
Fibromyalgie	○	○	○
Periphere arterielle Verschlusskrankheit (pAVK)	○	○	○
Sekundäre Kopfschmerzen	○	○	○
Migräne	○	○	○
Idiopathischer anhaltender Gesichtsschmerz	○	○	○
Neuralgien (z.B. Trigeminus)	○	○	○
Rheumatoide Arthritis	○	○	○
Multiple Sklerose	○	○	○

D02 [M per row]

Welche der nachfolgenden WHO-III-Opioide verordnen Sie derzeit zur Behandlung von **chronischen, nichttumorbedingten Schmerzen** und in welchen Darreichungsformen?

**WHO-III-Opioide (Wirkstoffgruppe)**	**Orale retardierte Darreichungsform**	**Orale (oder nasale) nichtretardierte Darreichungsform**	**Transdermale Darreichungsform**	**Diese WHO-III-Opioide verordne ich überhaupt nicht**
Morphin	☐	☐	*SCRIPTER: Please don't show this option*	○
Buprenorphin (z.B. Temgesic®, s.l.,Transtec®, Norspan®)	*SCRIPTER: Please don't show this option*	☐	☐	○
Fentanyl (z.B. Durogesic®, Effentora®)	*SCRIPTER: Please don't show this option*	☐	☐	○
Oxycodon (ggf. + Naloxon) (z.B. Oxygesic®, Targin®)	☐	☐	*SCRIPTER: Please don't show this option*	○

Hydromorphon (z.B. Palladon®)	☐	☐	*SCRIPTER: Please don't show this option*	○
Tapentadol (z.B. Palexia®)	☐	☐	*SCRIPTER: Please don't show this option*	○

D03 [M]

Bitte stellen Sie sich folgende Situation vor: Ein Patient mit chronischem, nichttumorbedingtem Schmerz und bereits länger bestehender Opioidverschreibung kommt mit der Bitte in Ihre Sprechstunde, die Opioiddosis zu erhöhen. Hinweise für einen interventionsbedürftigen Befund wie neu aufgetretene neurologische Störungen oder „red flags" gibt es nicht. Welche der nachfolgend beschriebenen Empfindungen haben Sie dabei schon einmal an sich selbst beobachtet (Mehrfachantworten sind möglich):

1 Ich fühle mich unter Druck gesetzt, die Dosis zu erhöhen.
2 Ich empfinde Hilflosigkeit, weil ich keine einfache Lösung anbieten kann.
3 Ich kann mit der Situation ganz gut umgehen.
4 Ich verspüre negative Emotionen wie Ärger und Wut.
5 Ich habe bei der Erhöhung der Dosis ein ungutes Gefühl.
6

Objektive medizinische Risikokompetenz (adaptierter „Critical Risk Interpretation Test"51 [CRIT])

Bevor Sie gleich evidenzbasierte Informationen zum Nutzen-Schaden-Profil einer längerfristigen Behandlung von Patient*innen mit chronischen, nichttumorbedingten Schmerzen mit WHO-III-Opioiden sehen, möchte wir Ihnen noch einige Fragen vorlegen, die sich nicht mit Opioiden im Speziellen beschäftigen, sondern die Fähigkeit untersuchen, wie gut man medizinische Informationen im Allgemeinen versteht. Täglich werden wir mit Informationen zu Nutzen und Schaden von Therapien, Medikamente und Krebsfrüherkennungen konfrontiert. Diese Informationen können in unterschiedlicher Form vermittelt werden. Einige Formulierungen und Zahlenformate ermöglichen es uns, den Nutzen und Schaden medizinischer Maßnahmen gut abzuschätzen. Andere hingegen wecken falsche Erwartungen. Mit den nachfolgenden fünf Fragen wollen wir untersuchen, wie Sie mit solchen Aussagen umgehen.

E01 [S]

Was denken Sie, welche der nachfolgenden Aussagen erlaubt es Ihnen am zuverlässigsten vorherzusagen, ob das neue Medikament besser ist als das alte Medikament? Bitte kreuzen Sie eine der Optionen an.

1 Eine große randomisierte Studie zeigte, dass von 33 Patient*innen, die das neue Medikament erhielten, nur einer verstarb.
2 Innerhalb der Patient*innen, die in einer großen randomisierten Studie ein neues Medikament erhielten, verstarben ca. 33% weniger als innerhalb der Patient*innen, die das alte Medikament erhielten.

3 Innerhalb der Patient*innen, die in einer großen randomisierten Studie ein neues Medikament erhielten, verstarben 6 von 100 Patient*innen. Innerhalb der Gruppe von Patient*innen, die das alte Medikament erhielten, waren es 9 von 100.
4 Ich weiß es nicht sicher.

E02 [S]

Was denken Sie, welche der nachfolgenden drei Aussagen erlaubt es Ihnen am zuverlässigsten zu bestimmen, ob eine Krebsfrüherkennung Nutzen für Ihre Patient*innen hat? Bitte kreuzen Sie eine der Optionen an.

1 Die Sterblichkeitsrate der Menschen, die an der Früherkennung teilnehmen, ist deutlich geringer als bei denen, die nicht an der Früherkennung teilnehmen.
2 Bei Menschen, die an der Früherkennung teilnehmen, werden deutlich mehr Tumoren entdeckt als bei denen, die nicht an der Früherkennung teilnehmen.
3 Die 5-Jahres-Überlebensrate ist bei Menschen, die an der Früherkennung teilnehmen, deutlich höher als bei denen, die nicht an der Früherkennung teilnehmen.
4 Ich weiß es nicht sicher.

E03 [S]

Bei einer 60-jährigen Frau wird vor einer Operation zur Sicherheit eine Testung auf eine AIDS-Erkrankung (HIV-Test) durchgeführt. Die Wahrscheinlichkeit, dass die Frau mit HIV infiziert ist, war vor der Testdurchführung sehr gering und lag bei ca. 1 in 100.000. Der Test fällt positiv aus, das heißt, das Testergebnis deutet auf eine HIV-Infektion hin. Der HIV-Test ist ein sehr zuverlässiger Test. Wenn jemand erkrankt ist, erkennt der Test das mit einer 99,9%igen Wahrscheinlichkeit richtig, wenn jemand nicht erkrankt ist, erkennt er dies ebenfalls mit einer 99,9%igen Wahrscheinlichkeit richtig. Was denken Sie, welche der nachfolgenden Aussagen beschreibt am besten, wie sicher es nach dem positiven Testergebnis ist, dass die Frau tatsächlich eine HIV-Infektion hat? Bitte kreuzen Sie die Aussage an, von der Sie denken, dass sie richtig ist.

1 Es ist sicher, dass die Frau HIV hat.
2 Es ist eher unwahrscheinlich, dass die Frau HIV hat.
3 Es ist völlig ausgeschlossen, dass die Frau HIV hat.
4 Ich weiß es nicht sicher.

E04 [S]

Welche der nachfolgenden Aussagen beweist am besten, dass das damit beschriebene neue Medikament tatsächlich einen höheren Nutzen für Patient*innen hat?

1 Eine große randomisierte Studie zeigte, dass Patient*innen mit dem neuen Medikament deutlich seltener einen Schlaganfall hatten als Patient*innen mit dem alten Medikament.
2 Eine große randomisierte Studie zeigte, dass der Cholesterinspiegel, der auf ein erhöhtes Schlaganfallrisiko hinweisen kann, bei Patient*innen mit dem neuen Medikament deutlich geringer war als bei Patient*innen mit dem alten Medikament.
3 Eine große randomisierte Studie zeigte, dass Patient*innen mit dem neuen Medikament im Vergleich zu denen mit dem alten Medikament eine deutlich geringere Rate an erhöhten Risikofaktoren für einen Schlaganfall hatten.
4 Ich weiß es nicht sicher.

E05 [S]

In einer multizentrischen Studie, welche das Ziel hat, die Effektivität von zwei Diabetes-Früherkennungsprogrammen zu untersuchen, liegt der Cut-Off für einen auffälligen Nüchternblutzucker im Programm A bei ≥ 6 mmol/l und im Programm B bei ≥ 7 mmol/l. Das bedeutet:

1 Programm A wird mehr Falsch-positiv-Diagnosen haben als Programm B.
2 In beiden Programmen werden ca. genauso viele Patient*innen mit Diabetes diagnostiziert werden.
3 Programm B wird mehr Falsch-positiv-Diagnosen haben als Programm A.
4 Ich weiß es nicht sicher.

Vielen Dank für Ihre Teilnahme an der Befragung.

Sie haben das Ende der Befragung erreicht und können das Fenster schließen.

Project: ERONA—Doctors

Translation of the German version of the survey

[Note: The ERONA project consisted of questions for several subprojects. In the following, only the questions concerning the current study are listed.]

Dear doctors,

Thank you very much for your interest in this study.

Ipsos is an independent market research institute. On behalf of the Max Planck Institute for Human Development, we are conducting the study project "ERONA" (Experiencing the Risks of Overutilizing Opioids Among Patients with Non-Tumor Chronic Pain in Outpatient Care) - funded by the German Federal Ministry of Health. The project intends to better understand how physicians make treatment decisions regarding strong opioids and how they view their effects.

In the future, the findings from this project will hopefully help to inform the presentation of medical risks in order to support physicians' treatment decisions.The Max Planck Institute would therefore be grateful if you participated in this online survey questionnaire. The survey will take about **30 minutes** and is incentivised. Before you begin, we would like to remind you that your participation is absolutely voluntary. You can terminate your participation at any time without giving reasons and without any disadvantages.

Please also be assured of the following:

- *We comply with all applicable laws in Germany concerning the protection of your personal data as well as the guidelines of the European Society for Opinion and Market Research (ESOMAR) and the Working Group of German Market and Social Research Institutes (Arbeitskreis Deutscher Markt- und Sozialforschungsinstitute e.V.) (ADM). You can obtain the data protection declaration for this study by clicking* here.
- *Your responses will be used by both us and the commissioning organisation for research purposes only.*
- *Your responses will be pooled together with those of the other participants and presented to the commissioning organisation in aggregated and anonymised form.*

If you wish to take part in the survey and agree to these conditions, please click "I agree" at the end of this page. Otherwise, leave the survey without any further actions.

To navigate through the survey, you will need to click the "Continue" button at the end of each page.

If this button is not visible, please scroll down. If you are unable to proceed to the next page, you may have missed a question on the page. In this case, please look for an error message and scroll down.

The ERONA project is a joint project of the Max Planck Institute for Human Development Berlin, Charité – Universitätsmedizin Berlin, the Robert Koch Institute Berlin, and the Institute for Evidence in Medicine (for Cochrane Foundation Germany), Freiburg.

We thank you in advance for your cooperation.

To ensure that our findings are as valid as possible, we need to make sure that the topic of the study is indeed relevant to all participants. The following questions—known as screener questions—serve to identify these people. Please answer each of the following screener questions. Depending on whether you belong to the target group for this study, you will either continue directly on to the study itself or your participation will be terminated.

S01 [S]

Which of the following describes your work?

4 General practitioner (GP)
5 Specialist with an additional qualification in specialised pain therapy
6 I am neither a general practitioner nor do I have an additional qualification in specialised pain therapy

S02 [O]

What is your medical specialty?

___

S03 [S]

Do you prescribe opioids falling under the Betäubungsmittelgesetz (BtM)[1] (e.g., morphine, Sevredol®, buprenorphine, Norspan®, Transtec®, Temgesic®, fentanyl, Durogesic®, oxycodone, Oxygesic®, Targin®, hydromorphone, Palladon®, Jurnista®, tapentadol, Palexia®[2]) for the treatment of patients with chronic noncancer pain?

---

[1] The Betäubungsmittelgesetz (BtM) is the German equivalent of the Controlled Substances Act. A special yellow BtM prescription is required for all of the strong opioids targeted in this study.
[2] These are the German brandnames.

3 Yes
4 No

Welcome to the ERONA study! Before we start with specific questions about the prescription of strong opioids for the treatment of patients with chronic noncancer pain, we would first like to assess some demographic information.

Please indicate your gender.

4 female
5 male
6 diverse

A02 [S]

Please select your age group.

6 < 20 years
7 20-39 years
8 40-59 years
9 60-79 years
10 ≥ 80 years

A03 [S]

How many years have you worked in your profession?

6 < 10 years
7 10-19 years
8 20-29 years
9 30-39 years
10 ≥ 40 years

A04 [S]

Where are you primarily based?

5 in a surgery/practice
6 in a medical care centre
7 in a hospital
8 in a rehabilitation/nursing facility

A05 [S]

In which region of Germany do you practice?

5 North [Schleswig-Holstein, Mecklenburg-Western Pomerania, Lower Saxony, Hamburg, Bremen]
6 South [Bavaria, Baden-Württemberg]
7 East [Saxony-Anhalt, Brandenburg, Saxony, Thuringia, Berlin]
8 West [Hesse, North Rhine-Westphalia, Rhineland-Palatinate, Saarland]

B01 [M per row]

Let us now take a closer look at strong opioids in the treatment of patients with chronic noncancer pain. Opioids can have positive and negative effects on your patients' health. Below are some of the positive and negative effects of using strong opioids to treat patients with chronic noncancer pain. For each effect listed, please indicate whether you have personally observed this effect in your patients or have only heard about it from guidelines, articles, studies, etc. If both options apply, please select both options. If neither option applies, please select "Neither." In other words, please make an entry in each line.

Possible effects of opioids	I have observed this in my patients	I have heard about this in guidelines, articles, studies, etc.	Neither
Reduction of pain	☐	☐	○
Improvement in ability to manage everyday life activities	☐	☐	○
Return to work	☐	☐	○
Increased fatigue	☐	☐	○
Decrease in cognitive abilities	☐	☐	○
Occurrence of dizziness	☐	☐	○
Occurrence of falls	☐	☐	○
Reduced ability to concentrate	☐	☐	○
Reduced affect display	☐	☐	○
Feeling of listlessness	☐	☐	○
Occurrence of constipation	☐	☐	○
Occurrence of sweating	☐	☐	○
Occurrence of nausea and vomiting	☐	☐	○
Difficulty urinating	☐	☐	○
Decreased libido	☐	☐	○
Continuous decrease in the effect of the opioids	☐	☐	○
Increase in pain	☐	☐	○
Intentional or unintentional use to reduce emotional tension	☐	☐	○
Signs of misuse (intentional)	☐	☐	○
Signs of incorrect use (unintentional)	☐	☐	○
Signs of opioid dependence	☐	☐	○

In the following, we would like to ask you questions that may tempt you to answer in a way that you think is more likely to meet with social approval. However, in order for our study to produce valid results, we need unbiased data. Your personal data are encrypted for this very reason: so that you can give the answer that best reflects your opinion without hesitation.

D01 [S per row]

For **which noncancer diseases** have you prescribed strong opioids as primary prescriber in the past 12 months? (Does not apply = did not have any patients with this condition)

**Condition**	**Yes**	**No**	**Does not apply**
Diabetic polyneuropathy	○	○	○
Chronic inflammatory bowel diseases (e.g., Crohn's disease, ulcerative colitis)	○	○	○
Cluster headache	○	○	○
Craniomandibular dysfunction	○	○	○
Postzoster neuralgia	○	○	○
Bedsores grade 3 and 4	○	○	○
Osteoarthritis	○	○	○
Non-specific back pain	○	○	○
Functional disorders	○	○	○
Intervertebral disc protrusion, prolapse	○	○	○
Fixed contractures	○	○	○
Primary headache	○	○	○
Spinal stenosis	○	○	○
Manifest osteoporosis with fractures of the spine	○	○	○
Chronic pancreatitis	○	○	○

Phantom pain	○	○	○
Chronic postoperative pain	○	○	○
Chronic regional pain syndrome (CRPS) Type I and II (Sudeck's disease)	○	○	○
Endometriosis	○	○	○
Brain lesion (e.g., apoplexy)	○	○	○
Fibromyalgia	○	○	○
Peripheral arterial occlusive disease (PAOD)	○	○	○
Secondary headache	○	○	○
Migraine	○	○	○
Persistent idiopathic facial pain	○	○	○
Neuralgia (e.g., trigeminal)	○	○	○
Rheumatoid arthritis	○	○	○
Multiple sclerosis	○	○	○

D02 [M per row]

Which of the following strong opioids do you currently prescribe for the treatment of **chronic noncancer pain** and in which dosage forms?

**Strong opioids (by active substance)**	**Slow-release oral dosage**	**Immediate-release oral (or nasal) dosage**	**Transdermal dosage**	**I do not prescribe these strong opioids at all**
Morphine	☐	☐	*SCRIPTER: Please don't show this option*	○
Buprenorphine (e.g., Temgesic® s.l., Transtec®, Norspan®)	*SCRIPTER: Please don't show this option*	☐	☐	○
Fentanyl (e.g., Durogesic®, Effentora®)	*SCRIPTER: Please don't show this option*	☐	☐	○
Oxycodone (where aproprIate + naloxone; e.g., Oxygesic®, Targin®)	☐	☐	*SCRIPTER: Please don't show this option*	○
Hydromorphone (e.g., Palladon®)	☐	☐	*SCRIPTER: Please don't show this option*	○

Tapentadol (e.g., Palexia®)	☐	☐	*SCRIPTER: Please don't show this option*	○

D03 [M]

Please imagine the following situation: A patient with chronic noncancer pain and a long-standing opioid prescription consults you requests an increase in the opioid dosage. There are no indications of findings requiring intervention, such as new neurological disorders or red flags. Which of the following have you ever observed in yourself in this situation (multiple responses are possible)?

7 I feel under pressure to increase the dosage.
8 I feel helpless because I cannot offer a simple solution.
9 I can handle the situation quite well.
10 I feel negative emotions like anger and rage.
11 I have a bad feeling about increasing the dosage.

Objective medical risk competence (adapted "Critical Risk Interpretation Test" 51 [CRIT]).

Before you see evidence-based information on the benefit–harm profile of long-term treatment with strong opioids for patients with chronic noncancer pain, we would like to ask you some questions that do not deal with opioids in particular, but examine the ability to understand medical information in general. Every day, we are confronted with information about the benefits and harms of medical treatments, medications, and cancer screening. This information can be conveyed in different ways. Some of the wordings and number formats enable us to make good assessments of the benefits and harms of medical interventions. Others raise false expectations. With the following five questions, we want to find out how you deal with this kind of information.

E01 [S]

Which of the following statements do you think enables you to predict most reliably whether the new medication is better than the older one? Please select one of the options.

5 A large randomised trial showed that out of 33 patients who received the new medication, only one died.
6 Of the patients who received a new medication in a large randomised trial, about 33% fewer died than of the patients who received the older medication.
7 Of the patients who received a new medication in a large randomised trial, 6 out of 100 patients died. In the group of patients who received the older medication, 9 out of 100 died.
8 I don't know for sure.

E02 [S]

Which of the following three statements do you think enables you to determine most reliably whether cancer screening is beneficial for your patients? Please select one of the options.

5 The mortality rate of people who participate in screening is significantly lower than that of those who do not.
6 Considerably more tumours are detected in people who participate in screening than in those who do not.
7 The 5-year survival rate is significantly higher in people who participate in screening than in those who do not.
8 I don't know for sure.

E03 [S]

A 60-year-old woman receives a screening test for HIV prior to surgery. Before the test was carried out, the probability she had HIV was very low (around 1 in 100,000). The test result is positive, that is, it indicates an HIV infection. The HIV test is a very reliable test. If someone has the disease, the test has a 99.9% probability of giving a correct result. If someone does not have the disease, the test also has a 99.9% probability of giving a correct result. Given the positive test result, which of the following statements do you think best describes how certain it is that the woman actually has HIV? Please select the statement you think is correct.

5 It is certain that the woman has HIV.
6 It is rather unlikely that the woman has HIV.
7 It is completely impossible that the woman has HIV.
8 I don't know for sure.

E04 [S]

Which of the following statements best proves that the new medication described indeed has greater benefits for patients?

5 A large randomised trial showed that patients on the new medication had considerably fewer strokes than patients on the older drug.
6 A large randomised trial showed that cholesterol levels, which can indicate an increased risk of a stroke, were significantly lower in patients on the new medication than in patients on the older medication.
7 A large randomised trial showed that patients on the new medication had a significantly lower rate of increased risk factors for a stroke than those on the older medication.
8 I don't know for sure.

E05 [S]

In a multicentre study aiming to investigate the effectiveness of two diabetes screening programmes, the cut-off for an abnormal fasting blood glucose is ≥ 6 mmol/l in programme A and ≥ 7 mmol/l in programme B. This means:

5 Programme A will have more false positive diagnoses than Programme B.
6 Approximately the same number of patients will be diagnosed with diabetes in both programmes.
7 Programme B will have more false positive diagnoses than Programme A.
8 I don't know for sure.

Thank you for participating in the survey.

You have reached the end of the survey and can close the window.
